# Chronic Pediatric Immune Thrombocytopenia Is Not Associated With Herpes Virus Infection Status

**DOI:** 10.3389/fped.2021.641535

**Published:** 2021-12-02

**Authors:** Tao Li, Gui-ling Yan, Zhu Luo, Qi Xie, Mei-mei Lai, Zhan-Guo Chen, Xiao-Qun Zheng

**Affiliations:** ^1^Department of Laboratory Medicine, The Second Affiliated Hospital and Yuying Children's Hospital of Wenzhou Medical University, Wenzhou, China; ^2^The Key Laboratory of Laboratory Medicine, School of Laboratory Medicine and Life Sciences, Wenzhou Medical University, Ministry of Education of China, Wenzhou, China

**Keywords:** chronic, children, herpes virus infections status, prognosis, ITP

## Abstract

**Background:** Immune thrombocytopenia (ITP) is characterized by non-chronic (transient, <12 months) and chronic (≥12 months) decline in the number of platelets. Herpes virus infections have been shown, in many studies, to be associated with the development of ITP. However, it remains unclear whether the herpes virus infection status is associated with the chronic ITP.

**Methods:** We reviewed 480 primary pediatric patients with ITP in the period from January 2017 to December 2019. The prevalence of herpes virus antibodies including the Cytomegalovirus (CMV), Herpes simplex virus 1 (HSV-1), Herpes simplex virus 2 (HSV-2), and Epstein Barr virus were recorded. The levels of serum complement C3 and C4, T (CD3+, CD4+, CD8+), B (CD19+) lymphocytes, and natural killer (CD16+ 56+) cells were also analyzed. Multivariate analysis was used to evaluate the associations between chronic ITP and herpes virus infection status.

**Results:** Compared with non-chronic, patients with chronic ITP had older age (≥3 years), lower levels of hemoglobin and complement C3, and lower probability of CMV and HSV-2 infections (IgM positive; *p* < 0.05). Patients with herpes virus infection had lower serum platelet counts (*p* < 0.001), lower complement C3 levels and lower CD4+/CD8+ cells ratio (*p* < 0.05). Furthermore, platelet counts were positively correlated with CD4+/CD8+ cells ratios (*r* = 0.519; *p* = 0.0078), and negatively correlated with T cells (CD3+: *r* = −0.458, *p* = 0.0213; CD8+: *r* = −0.489, *p* = 0.0131). Multivariate analysis showed that age (OR, 1.644; 95%CI, 1.007–2.684; *p* = 0.047) was an adverse risk factor for chronic ITP and CMV IgM positive (OR, 0.241; 95%CI, 0.072–0.814; *p* = 0.022) had lower risk of chronic ITP development, while other herpes virus infection statuses and clinical features were not.

**Conclusion:** Although herpes virus infections were associated with the onset of ITP, our findings indicated that herpes virus infection status might not be a risk factor for chronic ITP.

## Introduction

Immune thrombocytopenia (ITP) is an immunological disease of children and adults characterized by a transient or permanent decline in the number of platelets in their peripheral blood. The clinical characteristics are isolated thrombocytopenia and varying degrees of bleeding (1). Based on the etiology of the disease, it is divided into primary and secondary ITP owing to underlying autoimmune diseases, hematologic malignancies, use of certain medications, or acute infections (2, 3). According to the International Working Group (IWG) guidelines, ITP is defined as newly diagnosed when remission is within 3 months of diagnosis, persistent if remission occurs between 3 and 12 months after diagnosis and includes patients not reaching spontaneous remission or not maintaining a complete response off therapy, or chronic when it lasts for more than 12 months (1).

Most pediatric ITP patients could achieve complete recovery when treated with systemic steroids, intravenous immunoglobulin (IVIG), or anti-D immunoglobulin, and some achieve complete remission with no treatment (3–5). However, 10–20% of children were progressing to the chronic stage in long-term follow-up (3, 4). The prognosis of patients with ITP who were diagnosed with chronic ITP is not as good as those diagnosed with non-chronic (6). Several retrospective studies have investigated predictors for the development of chronic ITP (7–11), such as age, gender, and platelet counts. The Nordic group devised a weighted prediction score to evaluate a brief and uneventful course of newly diagnosed ITP in children (12). A recent study simplified the rules for diagnosing chronic ITP by analyzing 6–8 clinical features and identifying children as being of low, intermediate, and high risk for getting ITP (13).

With the understanding of the mechanism of ITP, it was considered to be an autoimmune disease (14). The presence of antibodies against platelets has traditionally been considered to play a central role in ITP. For example, autoantibodies against platelet glycoprotein (GP)IIb/IIIa or (GP)Ib/IX could be detected in the majority of patients (15, 16). Furthermore, the cellular immune responses of patients with ITP have the characteristic immune tolerance disorder (17). The immune function was mainly activated by T cells at diagnosis for ITP patients and they might have same immune status for chronic ITP (18, 19). However, a recent study showed that the level of kappa-deleting recombination excision circles (KRECs) was different in chronic patients with ITP compared to non-chronic patients. This points to an overreaction of the development of B-cells as a role in the pathogenesis of ITP (20).

The replication of the cytomegalovirus (CMV), and Epstein Barr virus (EBV) in the leukocytes of patients, may impact varying degrees on their immunological processes (21, 22). A primary viral infection is usually asymptomatic. However, viral latency in macrophages, T lymphocytes CD8+, and the endocrine cells may occur after viral infection. Several studies have suggested that the CMV and EBV may be common causes of autoimmune thrombocytopenia due to the phenomenon of antigenic mimicry which stimulates the production of antiplatelet antibodies (17, 23–25). In addition, primary ITP is more common in children and is usually triggered by non-specific viral infections, such as upper respiratory or gastrointestinal virus infections. In some cases, it is triggered by acute infection of EBV, CMV, Rubella virus (RV), Varicella, and Mumps (26, 27). These results may provide more information on the immune mechanism involved and an analysis of the prognosis. Viral infections may play a role in the occurrence of ITP. However, the association between related virus infections and the duration of ITP is still not clear.

Our present study aimed to evaluate the predictive value of clinical features and viral infections of childhood ITP. Furthermore, our study analyzed the differences in immune function between children with ITP and those with and without herpes virus infection.

## Patients and Methods

### Patients

A retrospective analysis of 480 children diagnosed with primary ITP between January 2017 and December 2019 at the Second Affiliated Hospital & Yuying Children's Hospital of Wenzhou Medical University, was conducted. Patients were diagnosed with ITP based having a platelet count of <100 × 10^9^/L, with no evidence of other known cause of thrombocytopenia, such as connective tissue disease, or malignancy, and drug induced thrombocytopenia (3). When treatment was considered necessary, patients were treated with corticosteroids, intravenous immunoglobulin (IVIG), or with only observation. Non-chronic (resolved within a year of diagnosis) and chronic (persisted for longer than a year after diagnosis) cases of ITP were defined according to the ASH 2011 Evidence-based Practice Guideline (3). Patients were followed-up with for at least 12 months after diagnosis.

#### Exclusion Criteria

**Figure 1:**
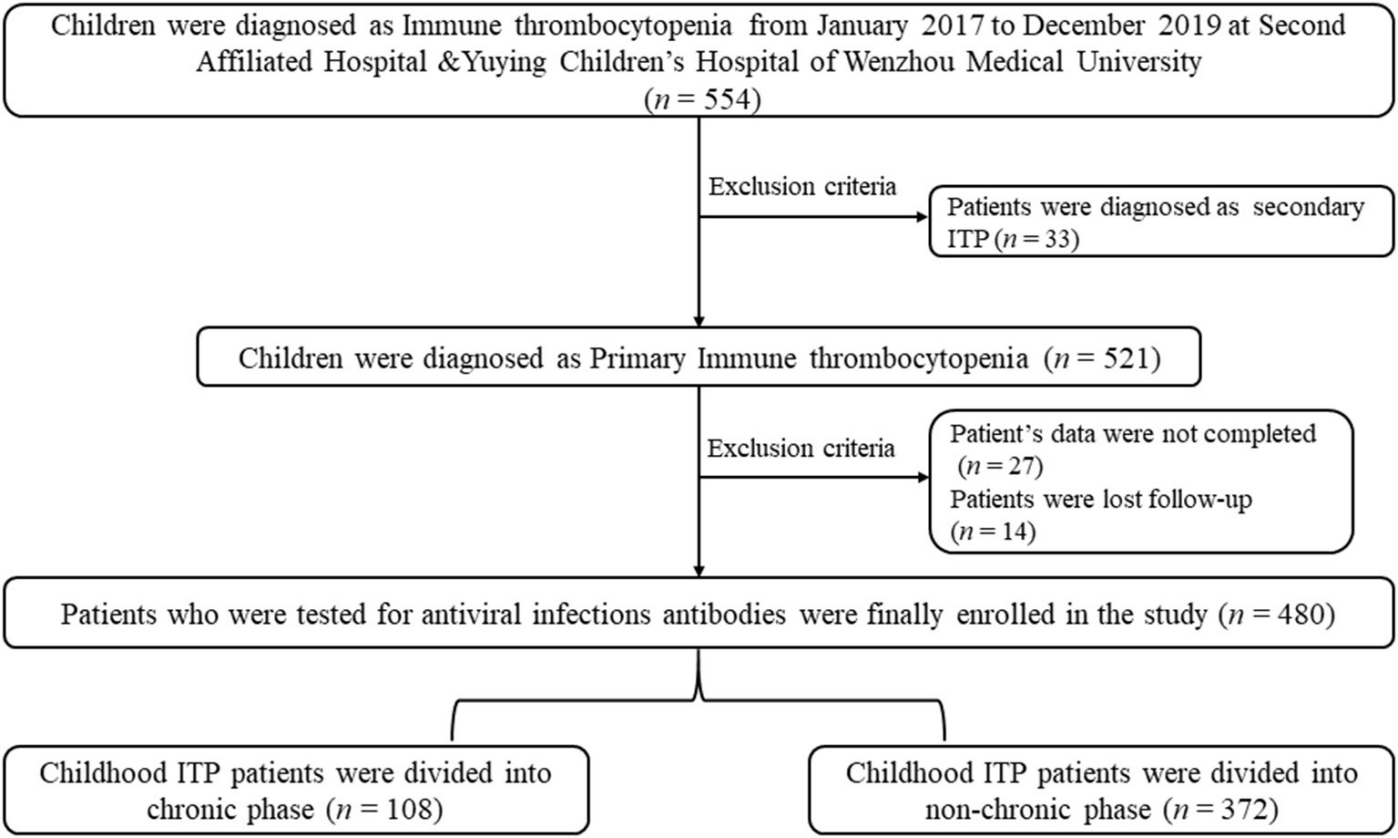
Study design and algorithm for deciding on which patients to include in our study.

Patients who were diagnosed with secondary ITP were excluded from the present study (*n* = 33). Also, patients who could not be located for follow-up (*n* = 27) and did not provide complete clinical data (*n* = 14) were excluded from the present study. The parents provided informed written consent for their children to participate in the present study. All patients signed informed consent. The present study was approved by the Research Ethics Boards of the Second Affiliated Hospital & Yuying Children's Hospital of Wenzhou Medical University and conducted following the Declaration of Helsinki. The design of study and algorithm for deciding on the inclusion of patients is illustrated in Figure 1.

The relevant information about each patient was collected from their medical records including age, gender, hepatomegaly, splenomegaly, bleeding manifestations, drug intake, diagnosis of onset (months), complete blood counts, hemoglobin, complement C3 and C4 levels, lymphocyte subsets, and defining duration of ITP. The manifestation of bleeding by patients was divided into epistaxis and mucocutaneous hemorrhaging. Patients were tested for CMV, Herpes simplex virus 1 (HSV-1), Herpes simplex virus 2 (HSV-2), the Epstein Barr virus (EBV), as well as IgG and IgM antibodies. Those clinical features and laboratory tested results were collected before treatment. The patients' detailed clinical information is summarized in Table 1.

**Table 1:** Clinical characteristics of the pediatric ITP patients.

**Variables**	**HC** **(*n* = 92)**	**Chronic** **(*n* = 108)**	**Non-chronic** **(*n* = 372)**	***p*-value**
Gender (*n*, %)				0.912
Male	56 (60.87)	65 (60.19)	221 (59.41)	
Female	36 (39.13)	43 (39.81)	151 (40.59)	
Age (years) (*n*), (%)				<0.001
0–3	27 (29.35)	42 (38.89)	226 (60.75)	
3–15	65 (70.65)	66 (61.11)	146 (39.25)	
Fever (*n*), (%)				0.997
Ever	NA	19 (17.59)	65 (17.47)	
Never	NA	89 (82.41)	307 (82.53)	
Hepatomegaly (*n*), (%)				0.037
Yes	NA	6 (5.56)	47 (12.63)	
No	NA	102 (94.44)	325 (87.37)	
Splenomegaly (*n*), (%)				1.000
Yes	NA	2 (1.85)	8 (2.15)	
No	NA	106 (98.15)	364 (97.85)	
Epistaxis (*n*), (%)				0.079
Yes	NA	11 (10.19)	20 (5.38)	
No	NA	97 (89.81)	352 (94.62)	
Diagnosis of onset (*n*), (%)				0.857
1–6 (months)	NA	57 (52.78)	200 (53.76)	
6–12 (months)	NA	51 (47.22)	172 (46.24)	
WBC counts (×10^9^/L), median (range)	NA	8.38 (3.5–25.2)	8.43 (1.45–24.6)	0.799
Hemoglobin (g/L), median (range)	NA	123.5 (85–157)	120 (44–159)	<0.001
PLT counts (×10^9^/L), median (range)		18 (1–87)	14 (1–85)	0.177
≤ 30 (n), (%)	NA	81 (75.00)	287 (77.15)	0.642
>30 (n), (%)	NA	27 (25.00)	85 (22.85)	
RBC counts (×10^12^/L), median (range)	NA	4.54 (3.38–5.44)	4.54 (1.57–6.37)	<0.001
Complement C3 (*n*), (%)				0.011
<90 mg/dl	NA	28 (27.78)	141 (43.28)	
90–180 mg/dl	NA	78 (72.22)	211 (56.72)	
Complement C4 (*n*), (%)	NA			0.251
<10 mg/dl	NA	4 (5.56)	24 (11.83)	
10–40 mg/dl	NA	102 (94.44)	328 (88.17)	
Initial treatment (*n*), (%)				0.686
IVIG	NA	84 (77.78)	296 (79.57)	
Observation or DEX	NA	24 (22.72)	76 (20.43)	

*IVIG, intravenous immunoglobulin; DEX, Dexamethasone. p value was compared chronic and non-chronic*.

### The Selection Criteria of Healthy Children

To ensure the accuracy and comprehensiveness of reporting in this case–control biomarker study, the present study complied with both the REMARK guidelines and the STROBE statement (ITP patients with herpes infection vs. HC). The level of complement C3 and C4 (*n* = 166; age, range: 3 months−15 years) were selected as the normal group from the department of Child health. Meanwhile, 92 healthy children (age; range: 4 months−14 years) who were tested for lymphocyte subsets were also collected. The selection criteria were followed: 1. These children had no history of active or chronic allergies, tumor or other upper respiratory disease. 2. All children signed informed consent.

### Complete Blood Count Test and Analysis

In the present study, each patient's CBC was obtained from peripheral venous blood using a Sysmex XI−5000, Japan. Blood cell counts at diagnosis were obtained prior to treatment with steroids and IVIG. PLT counts were converted to categorical variables according to the threshold 30 × 10^9^/L.

### Test for Antiviral Antibodies and Analysis

Screening for IgG and IgM antibodies against CMV and IgG antibodies against HSV-1 and HSV-2 were performed through the electrochemiluminescence method (Roche Cobas 8000, Germany). The Cutoff- Index (COI) and Concentration were used to express the results. IgM was defined as positive for CMV, and IgG was defined as positive for CMV, HSV-1, and HSV-2 when their respective COI: ≥1.0 U/ml. The IgG results for CMV are presented as the concentration. However, screening for IgG and IgM antibodies against the Epstein Barr virus (EBV), and for IgM antibodies of HSV-1, and HSV-2 were tested for by using chemiluminescence (YHLO Iflash 3000, Shenzhen China). The EBV kit uses three different populations of beads to screen for Abs directed against EBV nuclear antigen (EBVNA), EBV viral capsid antigen (EBVCA), and EBV early antigen diffuse (EBV EA). The following thresholds were considered positive: EBVNA IgG ≥ 20.0 U/mL; EBCVA IgM ≥ 40.0 U/mL; EBVCA IgG ≥ 20.0 U/mL; EBVEA IgG ≥ 20.0 U/mL; EBV EA IgM, HSV-1, and HSV-2 IgM positive, and included the COI ≥ 1.1.

### Complement C3 and C4 Test and Analysis

Complement C3 and C4 were tested by using the scattering turbidimetry method (Siemens BNIISystem, Germany). The following thresholds were considered to be within the normal range: C3: 90–180 mg/dl; C4: 10–40 mg/dl. The levels of the complements outside of their normal ranges were considered to be complement anomalies.

### Lymphocyte Subsets Test and Analysis

The detection of CD3+, CD4+, CD8+, and NK cells in the isolated PBMCs were done using flow cytometric detection (BD FACS Canto II, USA). Samples were enumerated using fluorescein isothiocyanate (FITC)-labeled monoclonal anti-CD3+, phycoerythrin-Cy7 (PC7) labeled anti-CD4+, allophycocyanin (APC)-Cy7-labeled anti-CD8+, allophycocyanin (APC)-anti-CD19+, and phycoerythrin (PE)-labeled anti-CD15+56+ (Beijing Tongsheng Shidai Biotech Co, LTD, China). Fifty μl of PBMCs were incubated with 20 μl anti-CD3+, anti-CD4+, anti-CD8+, anti-CD19+ and anti-CD15+56+ antibodies, respectively, for 15–25 min at room temperature and in the dark. After incubation, the hemolytic agent was added to dissolve the remaining peripheral cells. After acquisition of 25,000 events in a lymphocyte gate, the percentage of lymphocyte subsets was assessed. The following thresholds were considered to be within the normal range: CD3+: 61.28–80.87%; CD4+: 26.38–50.29%; CD8+: 19.24–25.62%; CD4+/CD8+ ratio: 0.98–1.94; CD16+ 56+:5.99–25.62%; CD19+: 6.74–18.91%.

### Statistical Analyses

Statistical analyses were performed using the software SPSS V. 26.0 (SPSS; Chicago, IL, USA). The groups of ITP patients were characterized and compared using descriptive statistics. For categorical data, percentages are given, and where appropriate, with confidence intervals. Proportions were compared using the Chi-square tests. Associations were assessed by using odds ratios (OR) and the 95% confidence limits. Prognostic factors were evaluated by calculating the predictive values. The results were considered to be statistically significant at *p* < 0.05.

## Results

### Clinical Characteristics of Patients

Among the 480 patients (age; range 1 months−14 years) included in present study, 368 patients (76.67%) had PLT counts ≤ 30 × 10^9^/L, and in 112 patients (23.33%) it was >30 × 10^9^/L. In addition, most patients (*n* = 380; 79.16%) had selected IVIG, and 18 patients selected Dexamethasone as the initial treatment. Ultimately, 108 patients (22.5%) had progressed to the chronic ITP and 372 patients did not. Our data showed that chronic patients had higher ages (≥3 years, *p* < 0.001), hepatomegaly (*p* = 0.037), lower hemoglobin (*p* < 0.001), lower RBC counts (*p* < 0.001), and lower levels of complement C3 (*p* = 0.011) than non-chronic patients (Table 1). No differences were observed for gender, fever before admission, splenomegaly, epistaxis, diagnosis of onset (months), WBC counts, PLT counts, and the level of complement C4 (Table 1).

### CMV and HSV-2 Were Different in Chronic and Non-chronic ITP Patients

Among the 480 patients tested for antiviral infection antibodies (IgM and IgG), 65 patients were CMV IgM (+), six patients were HSV-1 IgM (+), 22 patients were HSV-2 IgM (+), 22 patients were EBVCA IgM (+), and 41 patients were EBVEA IgM (+). Details of the distribution of the various antiviral antibodies are shown in Supplementary Figure 1. In addition, an analysis of the percentage of patients in the chronic phase of the disease revealed that patients age ≥3 years were more likely to progress to the chronic phase (Supplementary Figure 2).

An analysis of the difference in the antiviral antibodies between the chronic and non-chronic patients revealed that the IgM-subtype directed at CMV (*p* < 0.001, Table 2), and the IgG-subtype directed toward HSV-2 (*p* = 0.005, Table 2) were significantly different. Other antiviral antibodies were not detected.

**Table 2:** Differences in herpes virus infection between chronic and non-chronic pediatric ITP patients.

**Anti-infections antibodies** **(IgM or IgG) (+) seropositive (–) seronegative**	**Chronic** **(*n* = 108)**	**Non-Chronic** **(*n* = 372)**	***p*-value**
CMV IgM (+)	3 (2.78)	62 (16.67)	<0.01
CMV IgM (**–**)	105 (97.22)	310 (83.33)	
CMV IgG (+)	100 (92.59)	349 (93.82)	0.69
CMV IgG (**–**)	8 (7.41)	23 (6.18)	
HSV-1 IgM (+)	2 (1.85)	4 (1.08)	0.61
HSV-1 IgM (**–**)	106 (98.15)	368 (98.92)	
HSV-1 IgG (+)	81 (75.00)	286 (76.88)	0.65
HSV-1 IgG (**–**)	27 (25.00)	86 (23.12)	
HSV-2 IgM (+)	4 (3.70)	18 (4.84)	0.76
HSV-2 IgM (**–**)	104 (96.30)	354 (95.16)	
HSV-2 IgG (+)	59 (54.63)	257 (69.09)	0.05
HSV-2 IgG (**–**)	49 (45.27)	115 (30.91)	
EBVCA IgM (+)	2 (1.85)	20 (5.38)	0.19
EBVCA IgM (**–**)	106 (98.15)	352 (94.62)	
EBVCA IgG (+)	91 (84.26)	305 (81.99)	0.55
EBVCA IgG (**–**)	17 (15.73)	67 (18.01)	
EBVNA IgG (+)	92 (85.19)	313 (84.14)	0.792
EBVNA IgG (**–**)	16 (14.81)	59 (15.86)	
EBVEA IgM (+)	5 (4.63)	36 (9.68)	0.098
EBVEA IgM (**–**)	103 (95.37)	336 (90.32)	
EBVEA IgG (+)	4 (3.70)	28 (7.53)	0.161
EBVEA IgG (**–**)	104 (96.30)	344 (92.47)	

### Complement C3, C4, and Lymphocyte Subsets Levels

**Figure 2:**
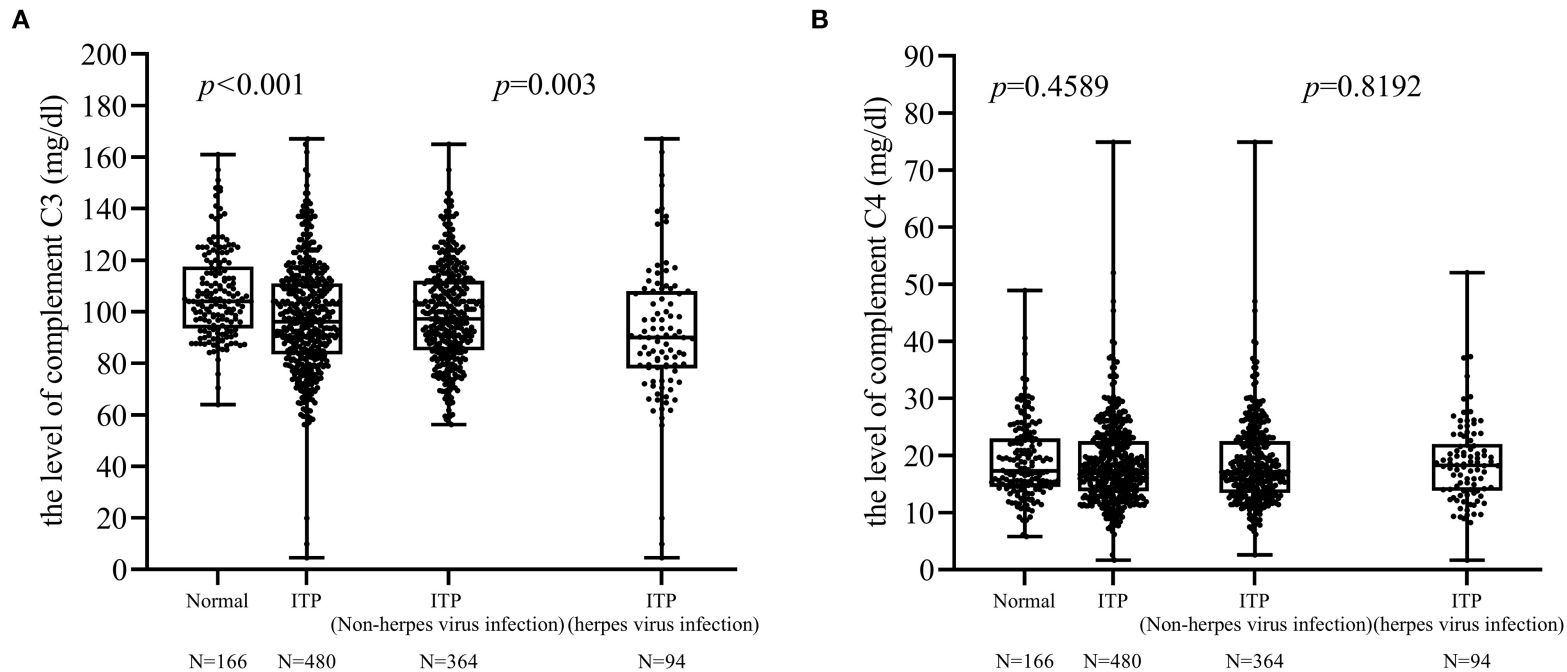
Differences in complement C3 and C4. **(A)** There was a difference in the complement C3 levels between ITP patients with and without herpes virus infection (*p* = 0.003). **(B)** No difference was found between these two groups in complement C4 levels.

Among the 458 patients tested for complement C3 and C4, there were 169 patients with abnormal complement function (range 4.53–89.9 mg/dl). In addition, we selected 166 healthy children (age, range: 3 months−15 years) whose complement C3 (range 64–161 mg/dl) and C4 (range 5.81–48.9 mg/dl) were also tested. Our data showed that a significant difference in the level of complement C3 between normal and children with ITP (*p* < 0.001). In addition, there was a significant difference in the complement C3 between ITP with herpes virus infection and without (*p* = 0.003; Figure 2A). There was no difference in complement C4 between these two groups (Figure 2B).

**Figure 3:**
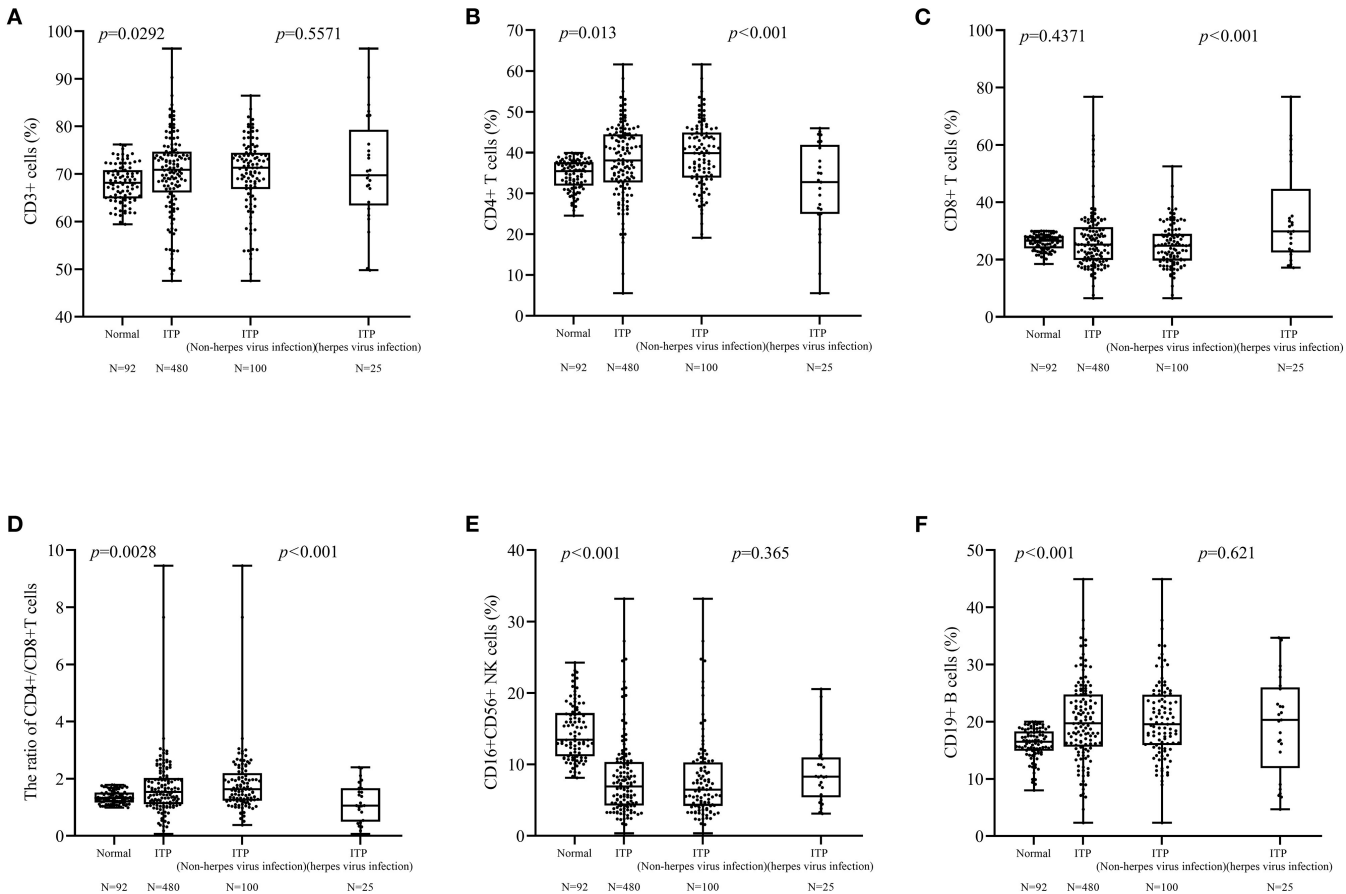
Differences between lymphocyte subsets. **(A,E,F)** Differences in CD3+, CD16+ 56+ cells were not found. **(B–D)** There were differences in the CD4+ cells (*p* < 0.001), CD8+ cells (*p* < 0.001), and CD4+/CD8+ cells ratio (*p* < 0.001) cells between patients with and without herpes virus infection.

Due to 14 patients with two or three herpes virus infections, the results of lymphocyte subsets were used once time. There were only 128 patients tested for lymphocyte subsets and a total of 22 patients (17.18%; age, 3 months−8 years) were positive for antiviral antibodies (IgM). Ninety-two healthy children (age, range: 4 months−14 years) were tested lymphocyte subsets. There was a significant difference in lymphocytes between healthy children (normal) and children with ITP (*p* < 0.01). There was significant difference between ITP with and without herpes virus infection in CD4+ (*p* < 0.001, Figure 3B), CD8+ (*p* < 0.001, Figure 3C), and CD4+/CD8+ ratio (*p* < 0.001, Figure 3D). There was no difference in the levels of CD3+, CD16+ 56+ and CD19+ cells between two groups (Figures 3A,E,F).

### The Correlation Between PLT Counts and Complement C3, C4, and Lymphocyte Subset Levels

**Figure 4:**
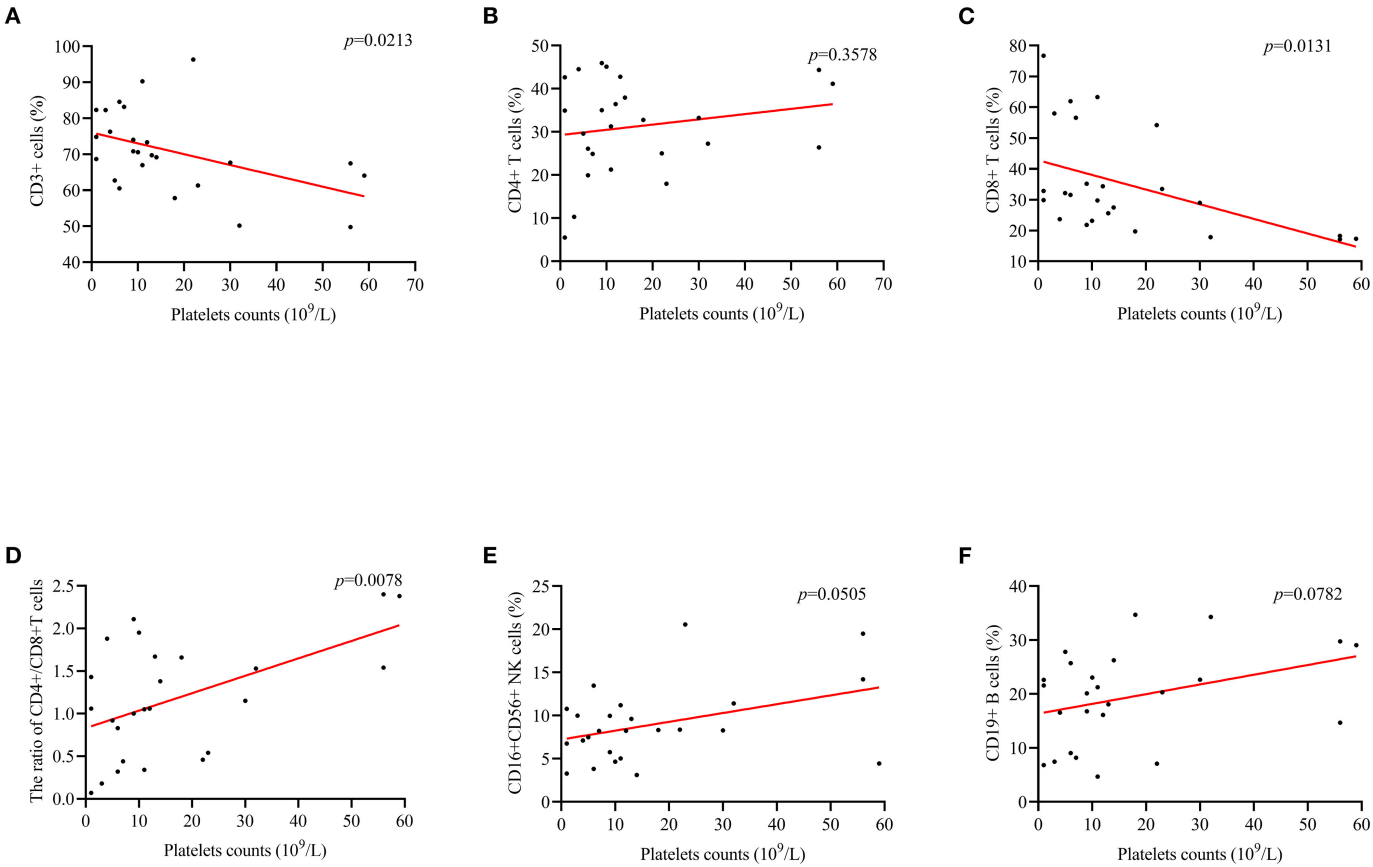
The correlation between PLT counts and the levels of lymphocytes in subset. **(D)** PLT counts were positively correlated CD4+/CD8+ cells ratio (*r* = 0.519; *p* = 0.0008). **(A,C)** There was a negative correlation between PLT counts and CD3+ (*r* = −0.458, *p* = 0.0213), and CD8+ (*r* = −0.489, *p* = 0.0131). **(B,E,F)** Differences in CD4+, CD16+ 56+, and CD19+ cells were not observed.

**Figure 5:**
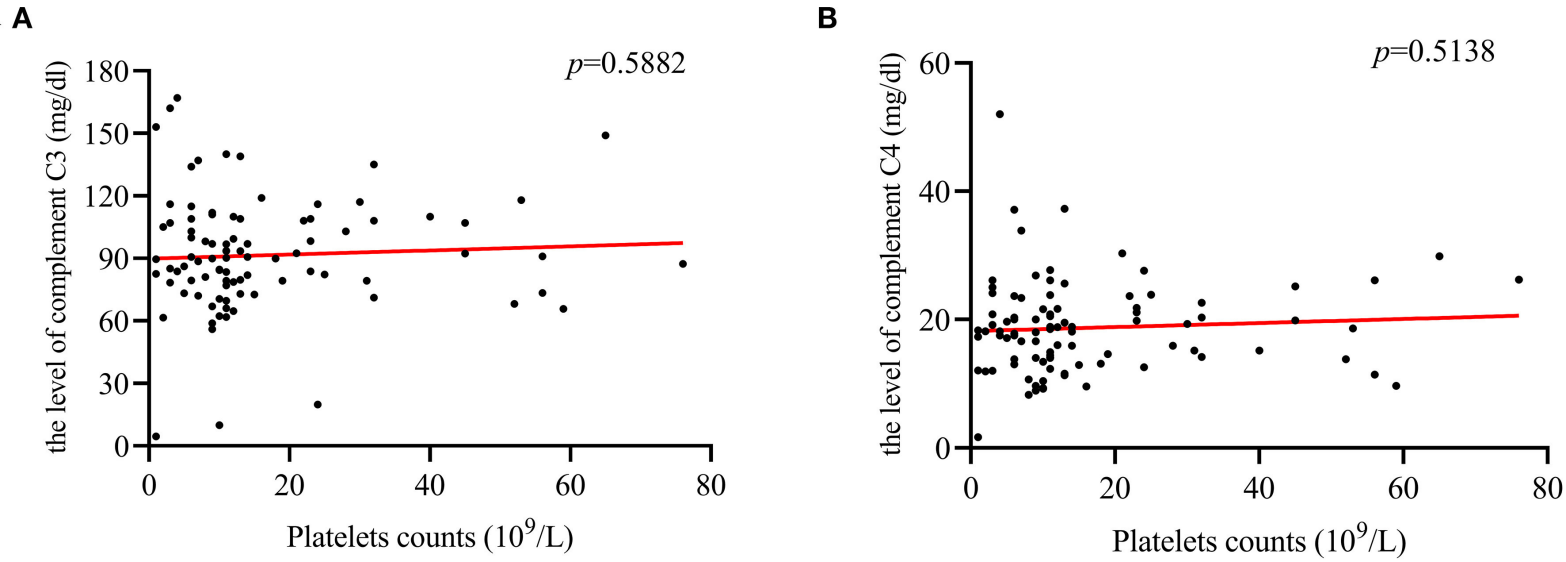
The correlation between PLT counts and complement C3, and C4 levels. **(A,B)** PLT counts were not correlated with the level of complement C3 (*r* = 0.09; *p* = 0.5882), or C4 (*r* = 0.094; *p* = 0.5138).

For ITP patients with herpes virus infection, a positive correlation between PLT counts and CD4+/CD8+ cells ratio (*r* = 0.519; *p* = 0.0078, Figure 4D) was found. In addition, the correlation between CD3+ cells (*r* = −0.458, *p* = 0.0213, Figure 4A), and CD8+ cells (*r* = −0.489, *p* = 0.0131, Figure 4C) was negative. However, there was no correlation with complement C3, C4, CD4+ cells and CD19+ cells (Figures 4B,E,F, 5A,B).

### Dynamic Changes of PLT Counts

**Figure 6:**
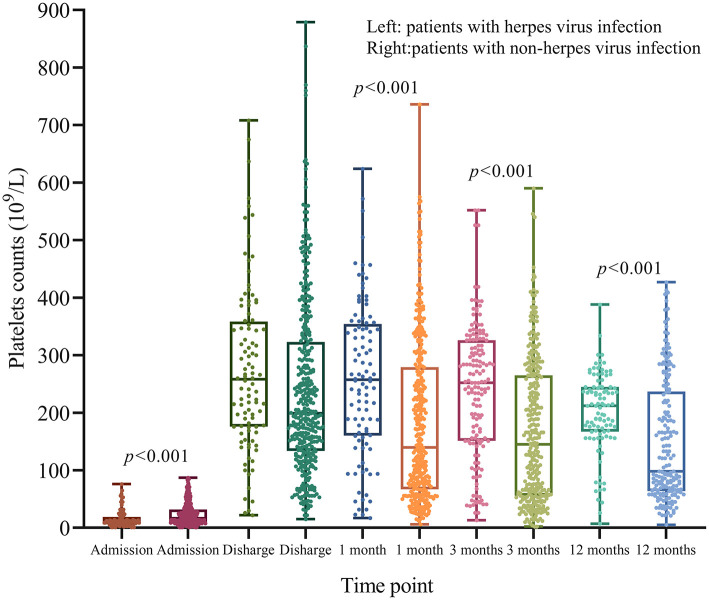
Dynamic changes of PLT counts after treatment. At admission, patients with herpes virus infection had lower PLT counts compared to those without herpes virus infection (*p* < 0.001). However, patients with herpes virus infection did not have lower PLT counts at 1, 3, and 12 months (*p* < 0.001).

At admission, ITP patients with herpes virus infection had lower PLT counts compared to patients without herpes virus infection (*p* < 0.001; Figure 6). However, patients with herpes virus infection had higher PLT counts at 1, 3, and 12 months after diagnosis (*p* < 0.01; Figure 6).

### Multivariate Analysis for Chronic ITP

The univariate analysis showed that patient' age (OR, 2.432; 95% CI, 1.586–3.774; *p* < 0.001), hepatomegaly (OR, 0.407; 0.169–0.979; *p* = 0.045), RBC counts (OR, 0.594; 95% CI, 0.356–0.992; *p* < 0.001), hemoglobin (OR, 1.037; 95% CI, 1.014–1.049; *p* < 0.001), RBC counts (OR, 1.858; 95% CI, 1.215–2.843; *p* = 0.004), low level of complement C3 (OR, 0.537; 95% CI, 0.332–0.869; *p* = 0.011), CMV IgM positive (OR, 0.143; 95% CI, 0.044–0.465; *p* = 0.001), and HSV-2 IgG positive (OR, 0.924; 95% CI, 0.887–0.963; *p* < 0.001) were associated with chronic ITP (Table 3). However, gender, fever before admission, splenomegaly, WBC counts, epistaxis (Ever), PLT counts, complement C4, and other antibodies of viral infections were not suitable predictors of chronic ITP (Table 3).

**Table 3:** Univariate and multivariate analyses for chronic ITP.

	**Univariate analysis**			**Multivariate analysis**		
**Variables**	**OR**	**95%CI**	***p*-value**	**OR**	**95%CI**	***p*-value**
Gender	1.033	0.667–1.599	0.885			
Age (≥3 years)	2.432	1.586–3.774	<0.001	1.644	1.007–2.684	0.047
Fever (Ever)	1.008	0.574–1.770	0.977			
Hepatomegaly	0.407	0.169–0.979	0.045	0.613	0.227–1.659	0.336
Splenomegaly	0.858	0.180–4.104	0.848			
Epistaxis (Ever)	1.996	0.925–4.308	0.078			
WBC counts	1.008	0.947–1.074	0.798			
Hb (g/L)	1.031	1.014–1.049	<0.001	1.015	0.988–1.043	0.280
PLT counts	1.008	0.997–1.018	0.169			
RBC counts	1.858	1.215–2.843	0.004	0.939	0.462–1.910	0.862
Complement C3	0.537	0.332–0.869	0.011	0.730	0.440–1.211	0.223
Complement C4	0.536	0.182–1.581	0.258			
CMV IgM	0.143	0.044–0.465	0.001	0.241	0.072–0.814	0.022
CMV IgG	0.824	0.358–1.898	0.649			
HSV-1 IgM	1.736	0.314–9.608	0.528			
HSV-1 IgG	0.995	0.981–1.008	0.456			
HSV-2 IgM	0.756	0.250–2.284	0.621			
HSV-2 IgG	0.924	0.887–0.963	0.000	0.781	0.487–1.252	0.304
EBVCA IgM	0.332	0.076–1.444	0.142			
EBVCA IgG	1.176	0.657–2.103	0.585			
EBVNA IgG	1.084	0.595–1.974	0.792			
EBVEA IgM	0.453	0.173–1.185	0.106			
EBVEA IgG	0.473	0.162–1.378	0.170			

The results of multivariate analysis showed that patients' age (OR, 1.644; 95 CI% 1.007–2.684; *p* = 0.047) which could as an adverse risk factor was correlated with chronic ITP. Although CMV IgM positive (OR, 0.241; 95%CI, 0.072–0.814; *p* = 0.022) was associated with chronic ITP, it might have lower risk of chronic ITP development. The correlation between chronic ITP and other variables were not found (Table 3).

## Discussion

Evaluating the chronic course of ITP is essential for establishing an appropriate management plan and searching for an underlying etiology. Several clinical factors have been investigated to identify patients who are more susceptible to becoming chronic (6, 12, 13, 28, 29). Our study suggested that age and low levels of hemoglobin were adverse risk factors for chronic ITP, which was similar to previous studies. As described previously, many viruses, such as CMV, EBV, and RV, are involved in the pathogenesis of ITP because they stimulate the production of antiplatelet antibodies (26, 27). Our data showed that patients with herpes virus infection had lower PLT counts compared to patients without herpes virus infection. However, our results showed that chronic ITP had lower proportion of CMV IgM and HSV-2 IgG and the multivariate analysis showed that patients with herpes virus infection had a low risk of progressing to become chronic. Those results suggest that herpes virus may be the trigger of ITP and the chronic phase was not associated with herpes virus infection status (HSV-2 IgG). The study by Smalisz-Skrzypczyk et al. showed that CMV or EBV infections are common in children with ITP and these infections do not seem to have an appreciable bearing on the clinical course and the response to treatment of children with ITP (26). Viral infections were identified solely based on laboratory tests, and only a few patients had clinical symptoms that could be associated with a viral infection. In addition, in our study we only tested for antiviral antibodies and did not obtain copies of the virus DNA from the pediatric ITP patients.

Our study showed that the CD4+/CD8+ cells ratio between patients with and without herpes virus infection was significantly different. The results suggest that children with viral infections will have more disordered immune function, especially T cell mediated immune responses. However, another study demonstrated that KRECs levels are different in chronic patients compared to non-chronic patients, pointing to an overreaction of the development of B cells as a role in the pathogenesis of ITP (20). Furthermore, the low level of complement C3 was also observed in patients with herpes virus infection. Enhanced peripheral complement activation has long been considered as one of the major pathogenic elements of immune thrombocytopenia (30, 31). The present study sheds light on complement activation in the pathogenesis of ITP (32). In conclusion, our study suggested that patients with herpes virus infection had higher immune dysfunction. However, herpes virus infection status may not be an adverse risk factor for transforming to the chronic in long-term follow up.

Although many studies have confirmed that patients with viral infections have an increased probability of an adverse prognosis (33–35), other studies have showed that there were no obvious interactions between viral infections and the burden of disease (36, 37). In our study, most children selected IVIG as their initial treatment which may not increase the probability of the disease going into remission and not progressing to the chronic phase. The recent study showed that the use of IVIG could not reduce the incidence of the chronic phase (38). However, the study by Tamminga et al. showed that there was a lower rate of chronic ITP after IVIG treatment (29). The results of difference in two studies may be the difference in the deadline for follow-up (6 vs. 12 months). Patients' age was adverse risk factors for patients with ITP, which is consistent with a previous study and shows that clinical workers must be more aware of the clinical features of the patients (8, 17).

We were also aware of some limitations in our study. Although viral load by PCR was a more robust way of assessing acute infection status, the detection of viral load was carried out in clinical laboratory until recent 2 years in our hospital and could not provide this critical data. This is the reason why the viral load was absent in this retrospective study. The results of single center retrospective cohort studies are not generalizable to other populations. The sample size of patients included in the present study was not large. In conclusion, our study explored the association between antiviral antibodies and chronic childhood ITP. Childhood ITP with herpes virus infection may not be an adverse risk factor, causing transformation to the chronic phase. It provides new insights into ITP therapy and management in children.

## Data Availability Statement

The raw data supporting the conclusions of this article will be made available by the authors, without undue reservation.

## Ethics Statement

The studies involving human participants were reviewed and approved by the Research Ethics Boards of the Second Affiliated Hospital & Yuying Children's Hospital of Wenzhou Medical University. The patients/participants provided their written informed consent to participate in this study. Written informed consent was obtained from the minor (s)' legal guardian/next of kin for the publication of any potentially identifiable images or data included in this article.

## Author Contributions

X-QZ: study design. TL and G-lY: statistical analysis and manuscript draft. ZL, QX, M-mL, and Z-GC: clinical data review and acquisition. All authors contributed to the article and approved the submitted version.

## Funding

This study was supported by grants provided Natural Science Foundation of Zhejiang Province, China (No. LY18H200006).

## Conflict of Interest

The authors declare that the research was conducted in the absence of any commercial or financial relationships that could be construed as a potential conflict of interest.

## Publisher's Note

All claims expressed in this article are solely those of the authors and do not necessarily represent those of their affiliated organizations, or those of the publisher, the editors and the reviewers. Any product that may be evaluated in this article, or claim that may be made by its manufacturer, is not guaranteed or endorsed by the publisher.
