# Supplementary material for: Chronic Pediatric Immune Thrombocytopenia Is Not Associated With Herpes Virus Infection Status
Source: Front Pediatr. 2021 Dec 2;9:641535. doi: 10.3389/fped.2021.641535 (PMC8678596; doi:10.3389/fped.2021.641535)
Supplement: Supplementary file 2 [file Data_Sheet_2.docx]

**Supplementary Figure 1** The distribution of antiviral antibodies in children with ITP

**Supplementary Figure 2** The percentage occurrence of chronic ITP in children of different ages
