# Supplementary figures and images for: Chronic Pediatric Immune Thrombocytopenia Is Not Associated With Herpes Virus Infection Status
Source: Front Pediatr. 2021 Dec 2;9:641535. doi: 10.3389/fped.2021.641535 (PMC8678596; doi:10.3389/fped.2021.641535)

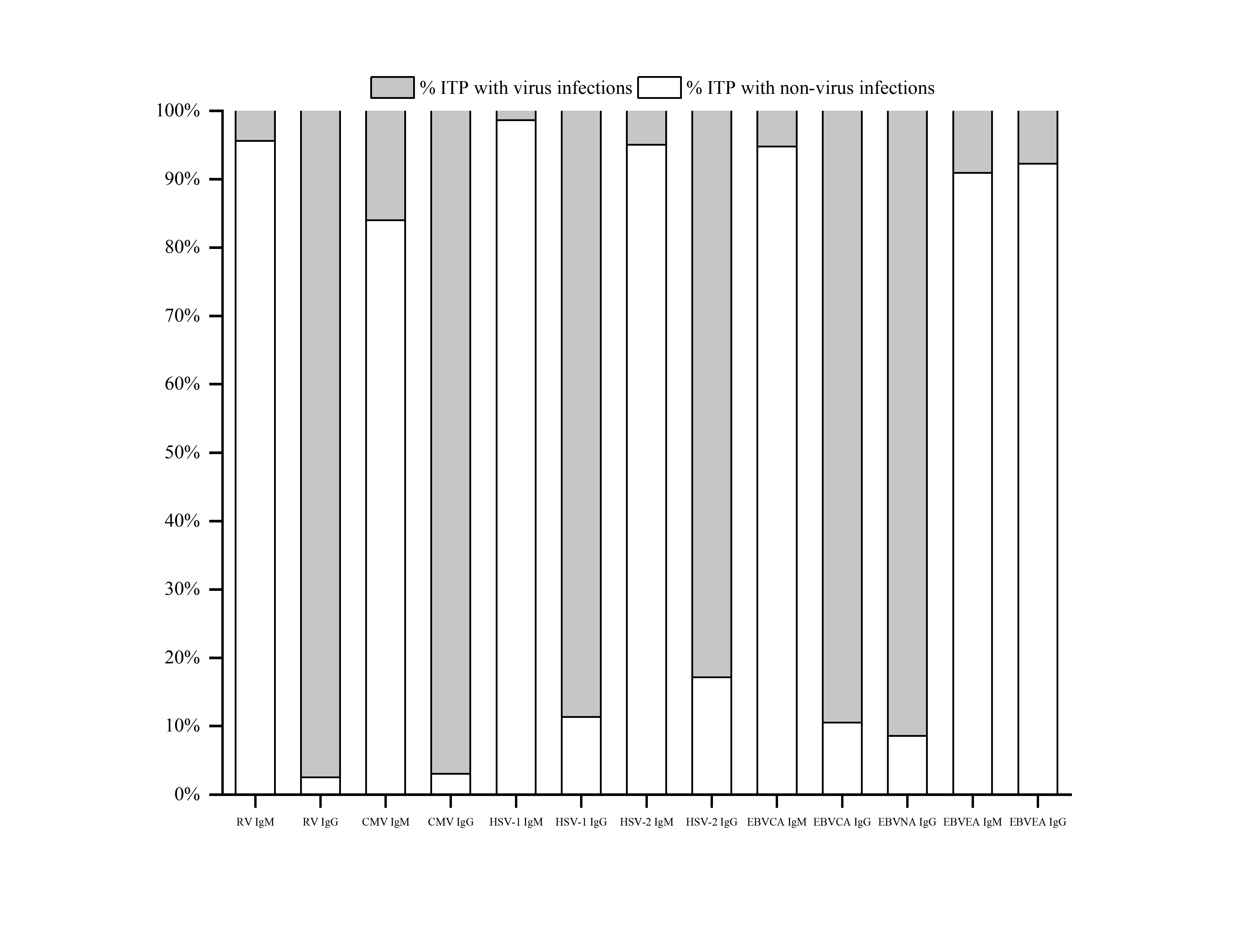

Supplement: Supplementary file 3 [file Image_1.TIF]

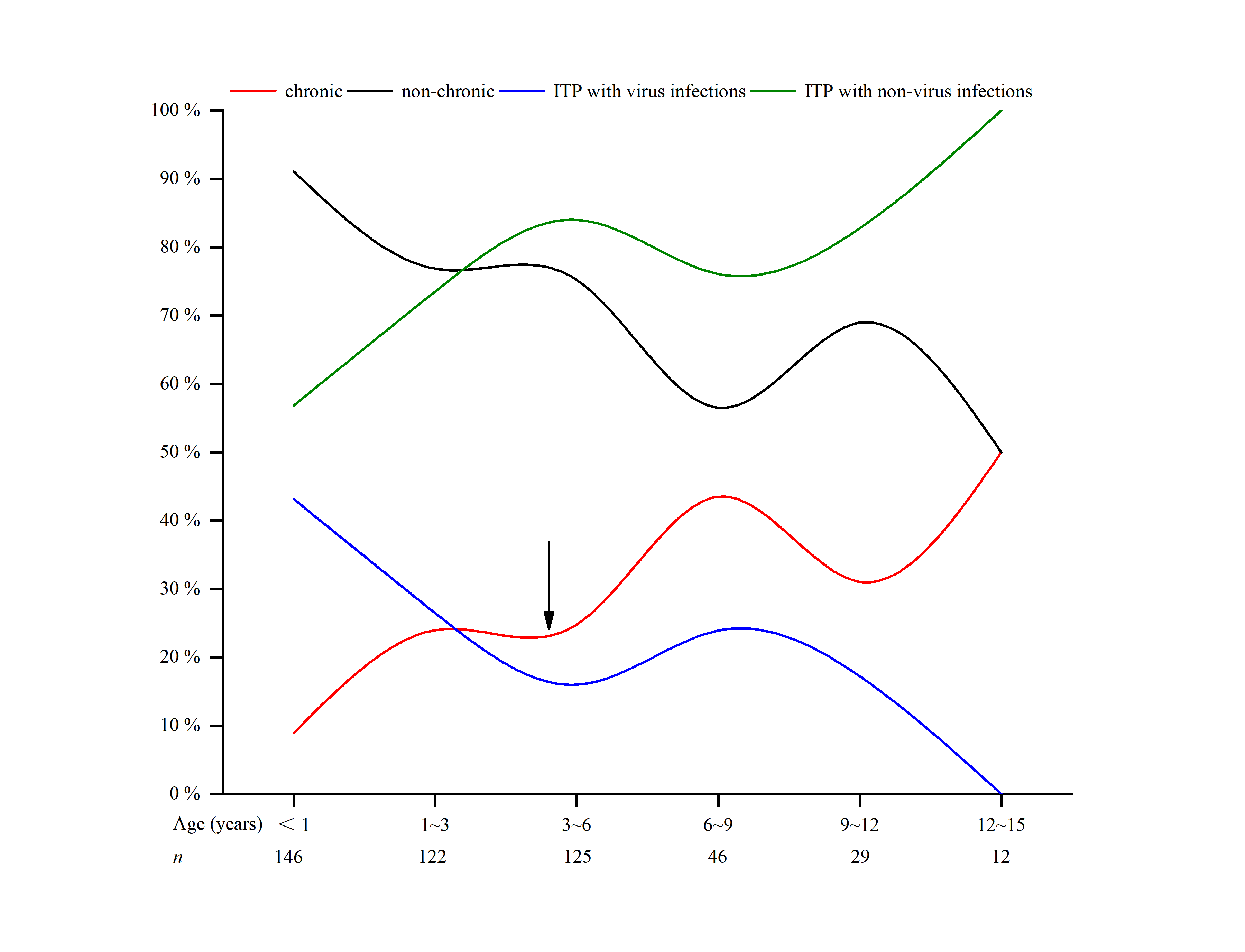

Supplement: Supplementary file 4 [file Image_2.TIF]
